# Metabolomics Coupled with Multivariate Data and Pathway Analysis on Potential Biomarkers in Cholestasis and Intervention Effect of *Paeonia lactiflora* Pall.

**DOI:** 10.3389/fphar.2016.00014

**Published:** 2016-02-04

**Authors:** Xiao Ma, Yong-Hui Chi, Ming Niu, Yun Zhu, Yan-Ling Zhao, Zhe Chen, Jia-Bo Wang, Cong-En Zhang, Jian-Yu Li, Li-Fu Wang, Man Gong, Shi-Zhang Wei, Chang Chen, Lu Zhang, Ming-Quan Wu, Xiao-He Xiao

**Affiliations:** ^1^Department of Pharmacy, 302 Military Hospital of ChinaBeijing, China; ^2^Pharmacy College, Chengdu University of Traditional Chinese MedicineChengdu, China; ^3^Cardiology Department, Beijing Chao-Yang Hospital, Capital Medical UniversityBeijing, China; ^4^China Military Institute of Chinese Medicine, 302 Military Hospital of ChinaBeijing, China; ^5^Department of Integrative Medical Center, 302 Military Hospital of ChinaBeijing, China

**Keywords:** *Paeonia lactiflora* Pall., cholestasis, metabolomics, bile acid secretion, biomarker

## Abstract

**Background:** The dried root of *Paeonia lactiflora* Pall. (PLP) is a classical Chinese herbal medicine that has been used to treat hepatic disease for 1000s of years. Our previous work suggested that PLP can be used to treat hepatitis with severe cholestasis. This study explored the mechanism by which PLP affects ANIT-induced cholestasis in rats using a metabolomics approach.

**Methods:** The effects of PLP on serum indices (TBIL, DBIL, AST, ALT, ALP, and TBA) and the histopathology of the liver were analyzed. Moreover, UHPLC-Q-TOF was performed to identify the possible effect of PLP on metabolites. The pathway analysis was conducted to illustrate the pathways and network by which PLP treats cholestasis.

**Result:** High-dose PLP remarkably down-regulated the serum indices and alleviated histological damage to the liver. Metabolomics analyses showed that the therapeutic effect of high-dose PLP is mainly associated with the regulation of several metabolites, such as glycocholic acid, taurocholic acid, glycochenodeoxycholic acid, L(D)-arginine, and L-tryptophan. A pathway analysis showed that the metabolites were related to bile acid secretion and amino acid metabolism. In addition, the significant changes in bile acid transporters also indicated that bile acid metabolism might be involved in the therapeutic effect of PLP on cholestasis. Moreover, a principal component analysis indicated that the metabolites in the high-dose PLP group were closer to those of the control, whereas those of the moderate dose or low-dose PLP group were closer to those of the ANIT group. This finding indicated that metabolites may be responsible for the differences between the effects of low-dose and moderate-dose PLP.

**Conclusion:** The therapeutic effect of high-dose PLP on cholestasis is possibly related to regulation of bile acid secretion and amino acid metabolism. Moreover, these findings may help better understand the mechanisms of disease and provide a potential therapy for cholestasis.

## Introduction

Cholestasis is a widespread disease that aﬄicts people worldwide due to its high morbidity. According to the reports from Centers for Disease Control, cholestasis is one of the top 15 causes of death in the United States (Yang et al., 2009). In China, this disease heavily burdens patients and society (Ma et al., 2014). Cholestasis is characterized by decreased bile flow and the concurrent accumulation of bile acids and is observed in a variety of hereditary and acquired liver diseases. It is one of the most common and devastating manifestations of liver disease and invariably is accompanied by a pool of outcomes without proper treatment, such as hepatic failure and cirrhosis (Zhao et al., 2013). It frequently occurs due to various endogenous and exogenous factors, but the progression of the disease seems to be similar (Delemos and Friedman, 2013). Oxidative stress, inflammation and the dysregulation of transporters are commonly thought to be the crucial pathological mechanism responsible for the development of this disease (Beuers et al., 2015). Moreover, recent metabolomics studies revealed that abnormal changes in antioxidative and cytoprotective metabolites as well as bile acids are also involved in this progression (Aoki et al., 2011; Beyoğlu and Idle, 2013).

Traditional Chinese medicine has been practiced in China for 1000s of years, and its application in the prevention and treatment of cholestasis has garnered increasing attention (Zhang et al., 2013; Chen et al., 2015b). The dried root of PLP [*Paeonia* species (Fam. Ranunculaceae), Chishao in Chinese], initially recorded in Shen Nong’s Chinese Materia Medica, has traditionally been used to reduce fever, eliminate stasis, activate blood circulation, and relieve pain based on the theory of TCM. In modern research, growing evidence indicates that PLP markedly affects liver diseases, such as acute liver injury, cholestatic hepatitis, and liver fibrosis (Li et al., 2011; Zhao et al., 2014, 2015). In our previous meta-analysis, high-dose PLP was effective in treating severe cholestasis (Ma et al., 2014). However, compared with studies of its clinical application and pharmacological effect, studies of the mechanism by which PLP alleviates cholestasis have been inadequate.

As a crucial component of systems biology, metabolomics depicts the whole metabolic profile by detecting 1000s of molecules in various biological fluids, such as the urine, saliva, and blood (Griffin, 2006). By analyzing specific early biomarkers during disease or drug treatment, metabolomics provides holistic insight into the relationship between a substance and metabolic pathways (Wang et al., 2011; Sun et al., 2012). Moreover, many studies revealed that metabolomics has increased the impetus to apply traditional medicine research in recent years (Cao et al., 2015). The Yinchenhao decoction, a famous Chinese formula for treating jaundice, has been proven effective in alleviating perturbations in multiple pathways in a jaundice model by a metabolomics analysis (Sun et al., 2014). Additionally, *Muntingia calabura* L., commonly used as a liver tonic in Southeast Asia, has been demonstrated to protect against CCl_4_-induced liver injury by affecting the biosynthesis of primary bile acid and metabolism of arachidonic acid, two major pathways, using an LC–MS metabolomics approach (Rofiee et al., 2015).

In this study, we performed a metabolomics assay using UHPLC coupled with Q-TOF mass spectrometry to characterize the metabolic profiles resulting from the effect of PLP on cholestasis. Furthermore, to deeply characterize this mechanism as a function of PLP dose, a systematic analysis of specific biomarkers and the unique biochemical pathways was conducted with multivariate data analysis techniques, which might provide novel insight into improving the treatment of cholestatic liver injury.

## Materials and Methods

### Water Extract of PLP Preparation

*Paeonia lactiflora* Pall. was purchased from Lvye, Co., Ltd. (Beijing, China). The origin and quality of PLP were identified according to the Chinese pharmacopeia (2015 Edition). A water extract of PLP was prepared by extracting the dried plant twice with water (2 h for the first extraction, 1.5 h for the second extraction). The extract was then decanted, filtered through six-layer gauze and evaporated to dry under reduced pressure. The final ratio of powder to raw herb was 25.8%. The extract was chemically characterized by LC–MS, and the main components in the water extract of PLP were identified as paeoniflorin, albiflorin, oxypaeoniflorin, and benzoylpaeoniflorin in our previous study (Ma et al., 2015).

### Animal Handling and Sample Preparation

#### Animal Handling

Male Sprague-Dawley rats weighing 200 ± 20 g were obtained from the laboratory animal center of the Military Medical Science Academy of the PLA [Permission No. SCXK-(A) 2012-0004]. All animals were allowed to acclimate for 1 week prior to the experiment and were kept at the same temperature (25 ± 2°C) and lighting (12:12 h light:dark cycle) conditions. Water and food were available to rats ad libitum. All studies were performed in accordance with the Guiding Principles for the Care and Use of Laboratory Animals of China. The animals were randomly divided into six groups of six rats each. The PLP water extract was dissolved in normal saline and intragastrically administered to rats at doses of 80, 40, or 20 g/kg⋅days for 5 days. Rats in the PLP groups intragastrically received 60 mg/kg ANIT (dissolved in olive oil) on the third day. The ANIT group received normal saline each day and was compared with the PLP groups. On the third day, the ANIT group was also treated with 60 mg/kg ANIT. The rats serving as a control received normal saline each day and intragastric treatment with the vehicle (olive oil) alone. UDCA, the positive control, was given to rats at dose of 60 mg/kg⋅days for 5 days using the same conditions used for the PLP or ANIT groups (Zhao et al., 2015).

#### Sample Preparation

The rats were sacrificed after the last treatment. Blood samples were collected and centrifuged at 3000 *× g* for 10 min to obtain serum. All serum samples were sterile, hemolysis-free and stored at -70°C before determining the biochemical parameters and conducting the metabolomics analysis. The serum levels of ALT, AST, TBIL, DBIL, TBA, and ALP were measured on a Synergy hybrid reader (Biotek, Winooski, VT, USA). The livers were excised and fixed in 10% PBS-buffered formalin. Three or four paraffin-embedded sections (4–5 μm thick) per specimen were prepared and stained with hematoxylin-eosin (HE staining). The stained sections were examined under a Nikon microscope (Tokyo, Japan) and analyzed using the image Pro-Plus 7200 software.

#### Western Blotting

Liver tissue (0.1 g) was homogenized and subsequently lysed in ice-cold lysis buffer containing 1 mM phenylmethylsulfonyl fluoride and a protease inhibitor mixture. The sample was centrifuged at 8000 × *g* and 4°C for 10 min to remove any debris. After centrifugation, the supernatant was aliquoted and stored at -80°C for the western blotting assay to detect BSEP, NTCP, and MRP2. Fifty micrograms of total liver protein was separated by 12% SDS-polyacrylamide gel electrophoresis and transferred to a nitrocellulose membrane. Immunodetection was performed using rabbit anti-BSEP antibody (1:1000), anti-NTCP antibody (1:1000), anti-MRP2 antibody (1:1000), and anti-beta ACTIN antibody (1:1000) in a solution of 5% milk in Tris-buffered saline and 0.05% Tween-20. After incubation with the appropriate secondary peroxidase-conjugated antibody, the membrane was washed in TBST for 60 min, and the immunoreactive bands were visualized with chemiluminescence.

### Metabolic Profiling

#### Injection Sample Handling

Two hundred microliters of thawed serum samples and 600 μL of methanol were transferred to a 1.5 mL polypropylene tube, and the solution was then mixed and allowed to stand for 20 min at 4°C before use. The samples were collected after centrifugation at 12,000 rpm and 4°C for 10 min to remove any solid debris. The supernatant was transferred to a polypropylene tube and then filtered through a syringe filter (0.22 μm) to obtain the injection samples.

#### Chromatography

Chromatography was performed using an Agilent 1290 series UHPLC system (Agilent Technologies, Santa Clara, CA, USA) equipped with quaternary pump, online degasser, autosampler, and thermostatted column compartment. The volume of injection samples was fixed at 4 μL. All samples were maintained at 4°C during the analysis. The separation was performed on a ZORBAX RRHD 300 SB-C18 column (2.1 mm × 100 mm, 1.8 μm, Agilent Technologies, Santa Clara, CA, USA). The column temperature was set to 30°C. The mobile phases consisted of 0.1% formic acid in acetonitrile (solvent A) and 0.1% formic acid in water (solvent B). The flow rate was set to 0.30 mL/min. The following gradient was used: a linear gradient of 95% A for the first minute, 95–60% A from 1.0 to 9.0 min, 60–10% A from 9.0 to 19.0 min, 10–0% A from 19.0 to 21.0 min, and 0% A from 21.0 to 25.0 min. The eluent was directly introduced to the mass spectrometer. After the injection of 10 samples, a pooled sample, the QC sample, followed by a blank was injected in order to ensure the stability and repeatability of the LC–MS systems.

#### Mass Spectrometry

For mass spectrometry, an Agilent 6550 Q-TOF/MS instrument (Agilent Technologies, Santa Clara, CA, USA) with an electrospray ionization source (ESI) in both positive and negative mode was used. Ionization was achieved using electrospray. The electrospray source parameters were fixed as follows: the electrospray capillary voltage was 3.0 kV in negative ionization mode and 4.0 kV in positive ionization mode. The mass range ranged from m/z 80 to 1000. The gas temperature was 200°C in negative ionization mode and 225°C in positive ionization mode. The gas flow was 11 L/min. The nebulizer was set to 35 pisg (negative) and 45 pisg (positive). The sheath gas temperature was 350°C, and the sheath gas flow was 12 L/min. The nozzle voltage was 500 V in both negative and positive mode.

#### Data Extraction

Sample data were extracted using the MassHunter Profinder software for peak detection and alignment. Full scan mode was employed in the mass range of 80–1000 amu. The initial and final retention times were set for data collection.

#### Multivariate Data Analysis

The resultant data matrices were introduced to the SIMCA-P 11.0 software (Umetrics, Umea, Sweden) for a PCA and PLS-DA. Prior to PCA, all variables obtained from the data matrix were mean-centered and scaled to a Pareto variance. PCA, an unsupervised pattern recognition approach, was used to reduce the dimension of LC–MS data and disclose intrinsic clustering of samples. To identify the variables responsible for this separation, the variable influence on the projection (VIP) parameter was used to select variables that most significantly contributed to the discrimination between the metabolomic profiles of the control, ANIT and PLP 80 g/kg groups in a PLS-DA model. VIP is a weighted sum of squares of the PLS weight, which indicates the importance of the variable to the entire model. Only variables with VIP values ≥ 1.0 were selected and used for further data analysis.

### Biomarkers Identification and Pathway Enrichment Analysis

Compounds with significant changes between groups (*p*-value < 0.05 and fold-change > 2) were selected as biomarkers. The potential biomarkers were identified by an online biochemical database service METLIN^1^. The recorded compound names and KEGG numbers were subjected to Metaboanalyst^2^ for further enrichment and a pathway analysis.

## Results

### Therapeutic Effects of PLP on Cholestasis Rats

As shown in **Table 1**, rats given ANIT displayed remarkable increases in the ALT and AST levels. Conversely, the serum levels of ALT and AST were significantly reduced when rats were treated with 80 and 40 g/kg PLP, respectively. The effect of 80 g/kg PLP was almost equal to that of UDCA. In addition, the level ALT but not that of AST was also altered by 20 g/kg PLP. The serum levels of TBIL, DBIL, ALP, and TBA were markedly enhanced in ANIT-treated rats compared with the control. UDCA (60 mg/kg) efficiently decreased the serum levels of TBIL, DBIL, and ALP but weakly decreased the serum level of TBA. Similarly, the levels of TBIL, DBIL, ALP, and TBA in rats with 80 and 40 g/kg PLP significantly decreased, which coincided with the findings of our previous study (Ma et al., 2015).

**Table 1:** Effect of PLP on serum ALT, AST, TBIL, DBIL, ALP, and TBA (*n* = 6, mean ± SD).

Group	ALT (IU/L)	AST (IU/L)	TBIL (μmol/L)	DBIL (μmol/L)	ALP (U/L)	TBA (μmol/L)
Control	15.78 ± 3.65	3.64 ± 0.81	6.09 ± 1.73	3.51 ± 0.89	59.21 ± 6.55	51.24 ± 6.88
ANIT	103.34 ± 12.18##	36.33 ± 5.61##	35.45 ± 4.97##	29.62 ± 3.45##	111.36 ± 11.95##	606.43 ± 50.98##
UDCA	69.61 ± 10.72^∗∗^	23.80 ± 4.74^∗∗^	19.53 ± 3.39^∗∗^	19.17 ± 4.66^∗∗^	89.34 ± 9.14^∗∗^	552.05 ± 52.67
PLP 80 g/kg	49.86 ± 10.27^∗∗^	15.99 ± 5.32^∗∗^	13.49 ± 3.40^∗∗^	17.69 ± 2.87^∗∗^	65.95 ± 10.39^∗∗^	443.39 ± 70.84^∗∗^
PLP 40 g/kg	60.75 ± 11.69^∗∗^	20.89 ± 3.22^∗∗^	15.91 ± 3.82^∗∗^	23.13 ± 4.04^∗∗^	69.44 ± 5.48^∗∗^	513.31 ± 66.22^∗∗^
PLP 20 g/kg	75.02 ± 15.44^∗∗^	38.32 ± 9.02	24.56 ± 4.10^∗∗^	25.94 ± 4.47^∗∗^	72.85 ± 8.16^∗∗^	568.80 ± 53.29

*Data were expressed as mean ± SD. ^∗∗^*p* < 0.01 compared with ANIT group; ##*p* < 0.01 compared with control group*.

**FIGURE 1:**
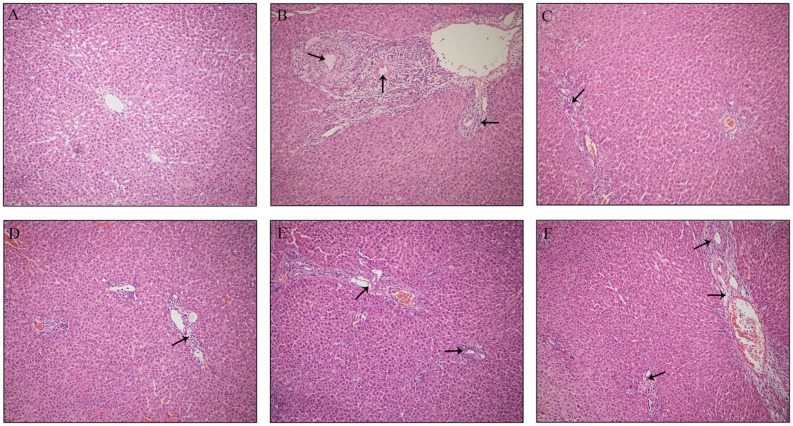
**Effect of PLP on histological changes in the liver tissue of ANIT-induced rats**. Hepatocytes damage was pointed by black arrows. **(A)** Control; **(B)** ANIT; **(C)** UDCA; **(D)** PLP 80 g/kg; **(E)** PLP 40 g/kg; **(F)** PLP 20/kg. (HE stained, 100× magnification).

Histological evaluations provided visual evidence for the protective effect of PLP on ANIT-induced cholestasis. As shown in **Figure 1A**, the hepatic tissues in the control group exhibited normal structures and were free of abnormal morphological changes in this study. The specimens from ANIT rats showed acute infiltration by polymorphonuclear neutrophils, edema, sinusoid congestion and hepatic necrosis (**Figure 1B**). Rats treated with UDCA and 80 g/kg PLP exhibited mild bile duct epithelial damage and hepatocyte hydropic degeneration as well as less neutrophil infiltration (**Figures 1C,D**). The specimens treated with 40 g/kg PLP exhibited moderate reductions in inflammatory cell infiltration and other ANIT-induced histological damage (**Figure 1E**). Specimens treated with 20 g/kg PLP exhibited almost no attenuation liver damage (portal tract edema, cholangitis, and bile duct epithelial damage; **Figure 1F**). These results indicated that 80 g/kg PLP continuously and significantly protected tissues from cholestasis.

### Multivariate Statistical Analysis and Potential Biomarkers Exploration

**FIGURE 2:**
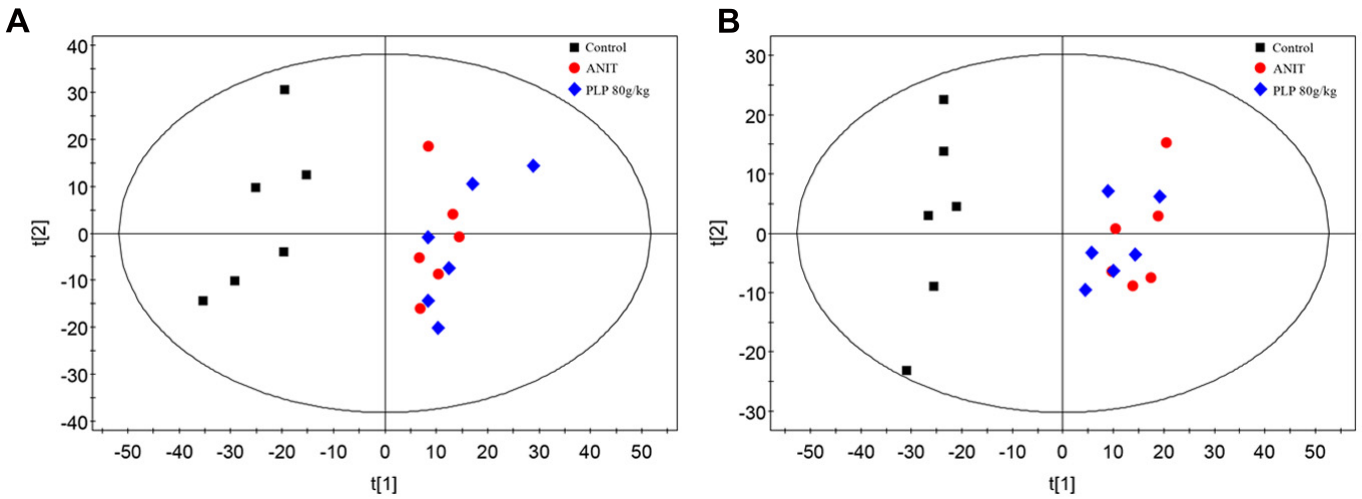
**Principal component analysis (PCA) score plot of control, ANIT and PLP 80 g/kg groups. (A)** ESI^+^ model; **(B)** ESI^-^ model.

Principal component analysis was initially used as an unsupervised statistical method to study differences in the metabolome between the control, ANIT and 80 g/kg PLP groups. A score plot provided a direct image of observational clusters. As shown in **Figures 2A,B**, the clustering significantly differed between the control and ANIT or 80 g/kg PLP groups in both positive and negative models. However, the clustering did not distinguish the ANIT and 80 g/kg PLP groups in PCA. The results of the PCA indicated that further multivariate statistical analysis was necessary to discern the relationship among these three groups.

**FIGURE 3:**
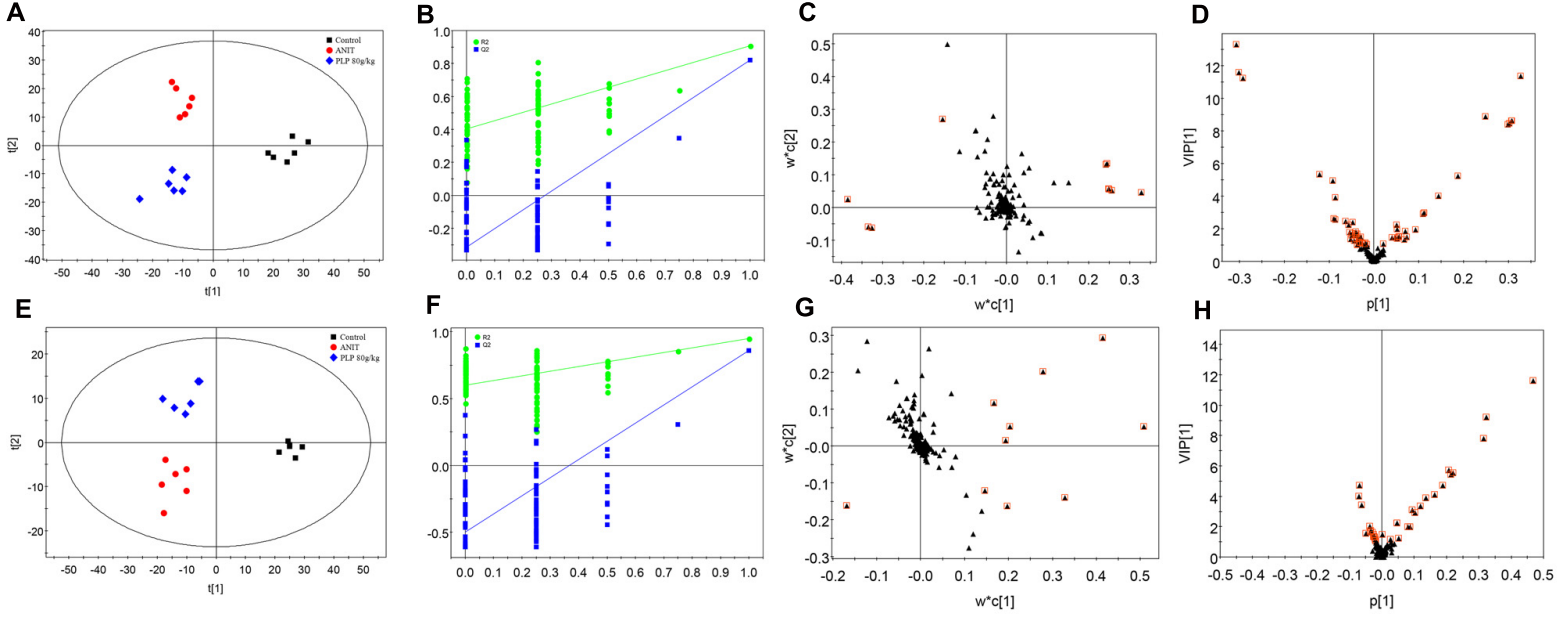
**Partial least-squares discriminant analysis (PLS-DA) of metabolomics data. (A)** Score plot in ESI^+^ model; **(B)** 100-permutation test in ESI^+^ model; **(C)** loading plot in ESI^+^ model; **(D)** VIP value plot in ESI^+^ model; **(E)** score plot in ESI^-^ model; **(F)** 100-permutation test in ESI^-^ model; **(G)** loading plot in ESI^-^ model; **(H)** VIP value plot in ESI^-^ model.

Partial least-squares discriminant analysis was then applied to further understand the different metabolomics patterns and identify potential biomarkers showing prominent concentration changes. As shown in **Figures 3A,E**, the clustering of the control, ANIT and 80 g/kg PLP groups could be discriminated in both positive and negative models. Commonly, the R^2^X, R^2^Y, and Q^2^ (cum) values provide an estimate of how well the model fits the data. In our positive model, the R^2^X, R^2^Y, and Q^2^ (cum) of PLS-DA were 0.750, 0.862, and 0.762, respectively. In addition, the R^2^X, R^2^Y, and Q^2^ (cum) of PLS-DA were 0.877, 0.856, and 0.740, respectively, in the negative model. These data indicated that models are of good quality and provide accurate predictions. Permutation tests with 100 iterations were performed to validate the model. These tests compared the goodness of fit of the original model with the goodness of fit of randomly permuted models. As shown in **Figures 3B,F**, the validation plots indicated that the original models were valid. The PLS-DA loading plots displayed variables that positively correlated with the score plots (**Figures 3C,G**). In our results, the loading plot demonstrated several crucial variables that were far from the center of the coordinate, indicating that these variables played an important role in clustering. We marked the top 10 variables with red box according to their VIP value (**Figures 3C,G**). Moreover, the VIP value plot was used to structurally identify these biomarkers (**Figures 3D,H**). In this work, a VIP value above 1.0 indicated by a red box was used as a screening standard to select potential metabolites.

### Identification of Potential Metabolites in Cholestasis Treatment

Among the 1979 signals detected in the control, ANIT and 80 g/kg PLP groups, variables that significantly contributed to the clustering and discrimination were identified according to a threshold of VIP ≥ 1.0. This threshold was obtained after PLS-DA processing these variables. According to the VIP, 903 variables were selected from the control, ANIT and 80 g/kg PLP groups as the candidates for fold-changes and ANOVA analyses. Next, candidates that significantly differed among the groups (the maximum and the minimum) with a *p*-value below 0.05 and fold-change exceeding two were identified as candidate biomarkers for METLIN and Metaboanalyst identification. Twelve potential biomarkers are summarized in **Table 2** with their corresponding retention time, m/z, formula, and differences by group.

**Table 2:** Identified metabolites of the serum from different groups.

No	R.T. (min)	Mass	Formula	Compounds	Ratio changes (significance)	
					Control/ANIT	PLP 80 g/kg/ANIT
1	0.85	174.113	C_6_H_14_N_4_O_2_	L(D)-Arginine	28.31^∗∗^	12.36^∗∗^
2	0.92	161.104	C_7_H_16_NO_3_	Carnitine	0.84	2.25^∗∗^
3	1.02	145.083	C_5_H_11_N_3_O_2_	4-Guanidinobutanoate	2.62^∗∗^	2.27^∗^
4	1.09	219.111	C_9_H_17_NO_5_	Pantothenate	0.45^∗∗^	0.71^∗∗^
5	3.95	204.088	C_11_H_12_N_2_O_2_	L-Tryptophan	0.98	0.44^∗∗^
6	4.93	135.068	C_8_H_9_NO	2-Phenylacetamide	2.77^∗^	14.67^∗∗^
7	10.22	346.212	C_21_H_30_O_4_	11-Deoxycortisol	3.64^∗∗^	1.97
8	10.50	465.306	C_26_H_43_NO_6_	Glycocholic acid	0.0047^∗∗^	0.18^∗∗^
9	10.58	449.311	C_26_H_43_NO_5_	Glycochenodeoxycholic acid	0.013^∗∗^	0.31^∗∗^
10	13.89	515.286	C_26_H_45_NO_7_S	Taurocholic acid	0.15^∗∗^	0.41^∗∗^
11	14.52	562.266	C_34_H_34_N_4_O_4_	Protoporphyrin IX	0.33^∗∗^	0.91
12	15.36	399.332	C_23_H_45_NO_4_	L-Palmitoylcarnitine	0.41^∗∗^	0.61^∗∗^

*^∗∗^*p* < 0.01, ^∗^*p* < 0.05 compared with ANIT group*.

### Pathway Analysis of Cholestasis Treatment

**FIGURE 4:**
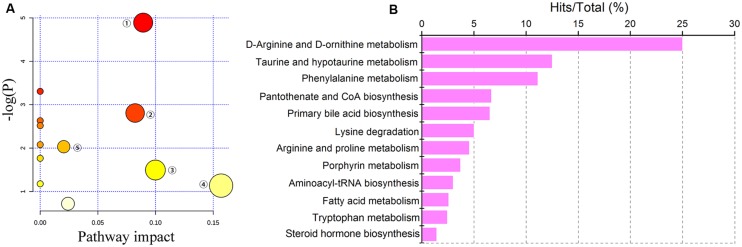
**Pathway analysis of cholestasis treatment. (A)** Bubble map of control, ANIT and PLP 80 g/kg groups in pathway analysis; ① primary bile acid biosynthesis; ② arginine and proline metabolism; ③ porphyrin and chlorophyll metabolism; ④ tryptophan metabolism; ⑤ pantothenate and CoA biosynthesis; **(B)** percentage of pathways among control, ANIT and PLP 80 g/kg comparison.

Detailed analyses of pathways were performed by Metaboanalyst, which is a free web-based tool that combines results from powerful pathway enrichment analyses with a topology analysis. The metabolic pathway analysis revealed that metabolites that were important for changes in cholestasis were responsible for D-arginine and D-ornithine metabolism, taurine and hypotaurine metabolism, porphyrin metabolism, pantothenate and CoA biosynthesis, primary bile acid biosynthesis, steroid hormone biosynthesis, arginine and proline metabolism, phenylalanine metabolism, lysine degradation, etc. (**Figures 4A,B**). As shown in **Figure 4A**, the top five pathways that impacted the bubble map were primary bile acid biosynthesis, arginine and proline metabolism, porphyrin and chlorophyll metabolism, tryptophan metabolism, and pantothenate and CoA biosynthesis. The top 5% of metabolite hits in pathways were D-arginine and D-ornithine metabolism, taurine and hypotaurine metabolism, porphyrin metabolism, pantothenate and CoA biosynthesis, and primary bile acid biosynthesis (**Figure 4B**).

### Signaling Networks Associated with the Pathways of Differentially Expressed Metabolites

**FIGURE 5:**
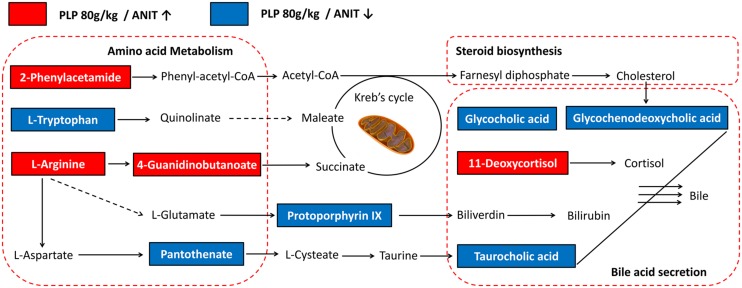
**Signaling networks associated with the differentially expressed metabolites pathways**. The red solid box represented as the peak area of PLP 80 g/kg/ANIT > 1; the blue solid box represented as the peak area of PLP 80 g/kg/ANIT < 1.

To reveal the interrelatedness among these signaling pathways, the identified metabolites and pathways were imported into the KEGG database to identify interactions. The networks were primarily related to amino acid metabolism, steroid biosynthesis, and bile secretion. According to the flow of the pathways, arginine, phenylalanine, and tryptophan metabolism were considered to be the up-stream signaling network. Moreover, glycocholic acid, taurocholic acid, and glycochenodeoxycholic acid were the direct constituents of bile and served as the down-stream signaling network. In addition, protoporphyrin IX and 11-deoxycortisol were components of bilirubin and cortisol, which are also essential substances of bile. Compared with the ANIT group, the levels of glycocholic acid, taurocholic acid, glycochenodeoxycholic acid, protoporphyrin IX, pantothenate and L-tryptophan were markedly down-regulated by 80 g/kg PLP compared with the control group, as indicated by decreases in the peak areas. Conversely, L(D)-arginine, 2-phenylacetamide, 4-guanidinobutanoate, and 11-deoxycortisol were significantly up-regulated in the 80 g/kg PLP group (**Figure 5**).

### Western Blotting for Bile Acid Metabolism Confirmation

**FIGURE 6:**
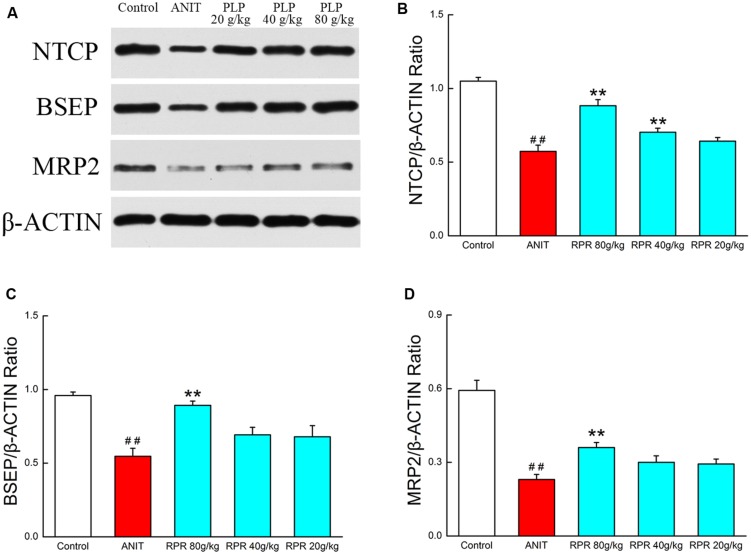
**Western blotting for bile acid metabolism confirmation. (A)** The western blot images of NTCP, BSEP, and MRP2; **(B)** NTCP protein level in liver tissue; **(C)** BSEP protein level in liver tissue; **(D)** MRP2 protein level in liver tissue. ^∗∗^*p* < 0.01, compared with ANIT group; ##*p* < 0.01, compared with control group.

To ensure that PLP primarily exerts its effect by regulating bile acid, we further explored the protein expressions of several transporters such as BSEP, MRP2, and NTCP. The results showed that the expression of BSEP, MRP2, and NTCP was markedly decreased in ANIT-treated rats compared with the control. Furthermore, treatment with 80 g/kg PLP significantly increased the low expression levels of NTCP, BSEP, and MRP2 in rats. However, these increases were limited in response to 40 and 20 g/kg PLP (**Figures 6A–D**).

### Potential Metabolite Changes in Cholestasis in Response to Treatment with Different Doses of PLP

**FIGURE 7:**
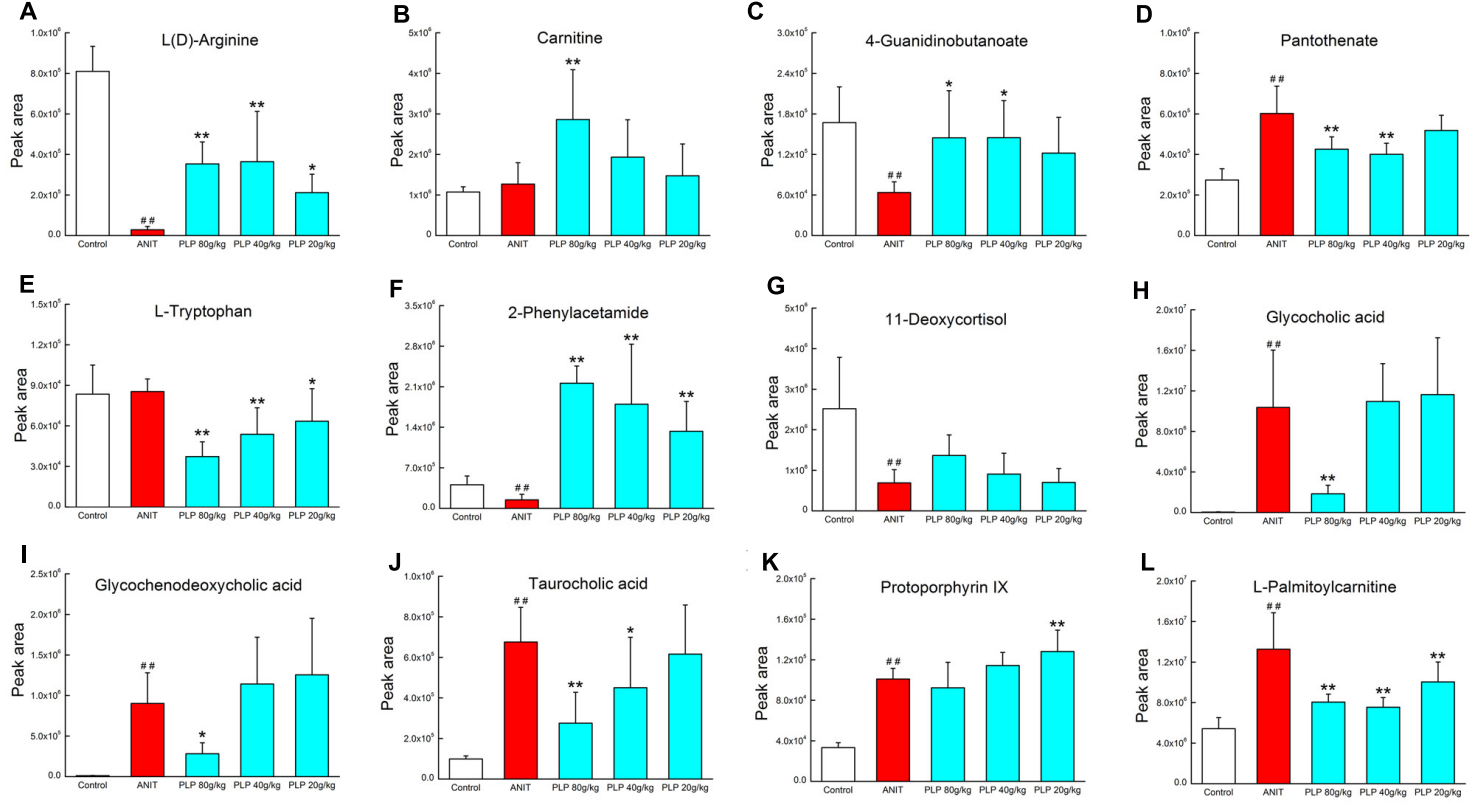
**Potential metabolites changes in cholestasis with different doses of PLP treatment. (A)** L(D)-Arginine; **(B)** carnitine; **(C)** 4-guanidinobutanoate; **(D)** pantothenate; **(E)** L-tryptophan; **(F)** 2-phenylacetamide; **(G)** 11-deoxycortisol; **(H)** glycocholic acid; **(I)** glycochenodeoxycholic acid; **(J)** taurocholic acid; **(K)** protoporphyrin IX; **(L)** L-palmitoylcarnitine. ^∗∗^*p* < 0.01, ^∗^*p* < 0.05 compared with ANIT group; ##*p* < 0.01 compared with control group.

Phenotype and histopathology analyses indicated that high-dose PLP (80 g/kg) significantly affects cholestasis. Conversely, only mild changes were observed in the 40 g/kg PLP group compared with the ANIT group, and low-dose PLP (20 g/kg) did not produce marked changes. Therefore, we analyzed changes in 12 potential metabolites in order to deeply characterize differences induced by treatment with various doses of PLP treatment. Compared with the ANIT group, 80 g/kg PLP significantly decreased the levels of glycocholic acid, taurocholic acid, and glycochenodeoxycholic acid (**Figures 7H–J**). However, 40 and 20 g/kg PLP produced milder changes in these metabolites, which indicated the limited regulation of bile acid synthesis or secretion (**Figures 7H,I**). The levels of L(D)-arginine, 4-guanidinobutanoate and 2-phenylacetamide increased, whereas the levels of L-tryptophan, pantothenate and L-palmitoylcarnitine markedly decreased in response to all three doses of PLP (**Figures 7A,C–F,L**). Among the three different PLP doses, the changes in metabolism were most pronounced in response to 80 g/kg PLP. Other potential metabolites, such as 11-deoxycortisol and protoporphyrin IX, did not exhibit marked changes in response to high-dose PLP (**Figures 7G,K**), and carnitine exhibited irregular changes in response to the three doses (**Figure 7B**).

### PCA of Potential Metabolites in Response to PLP Treatment

**FIGURE 8:**
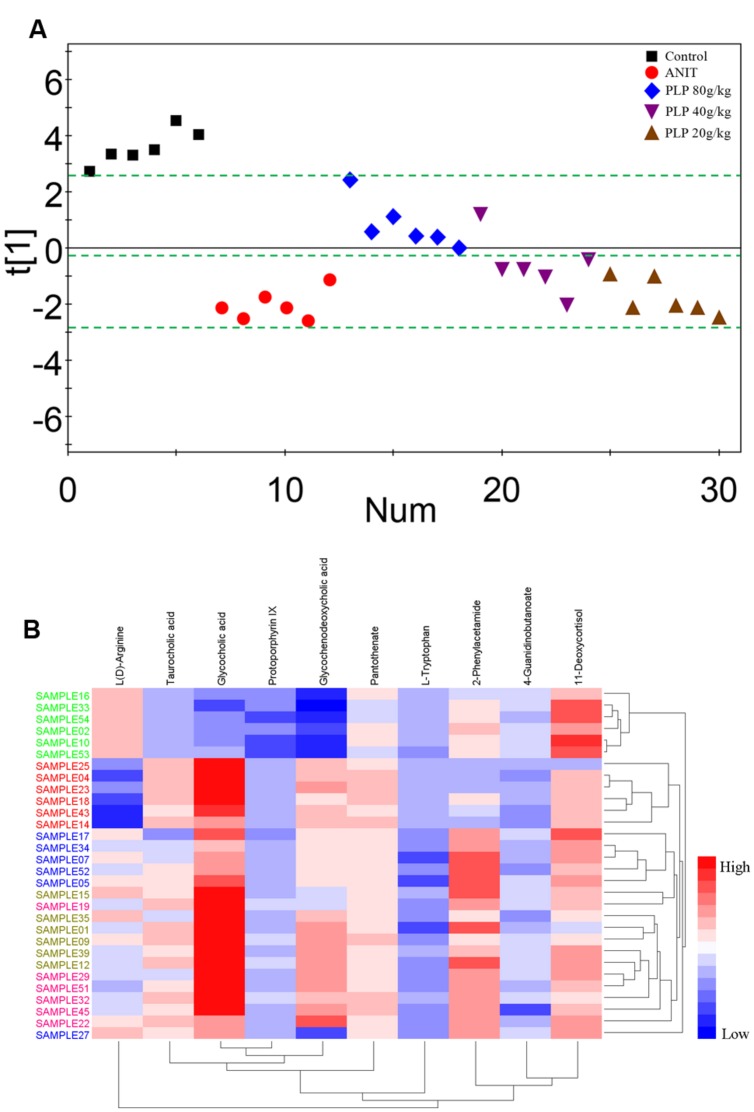
**Principal component analysis and heat map of potential metabolites among PLP treatment. (A)** One dimensional score plot of PCA with potential metabolites. The green bar classified the samples into four classes. X axis was the number of samples. **(B)** Heat map of potential metabolites. Red in gradient presented the increases. Blue in gradient presented the decreases. Samples with green was control group, samples with red was ANIT group, samples with blue was PLP 80 g/kg group, Samples with light brown was PLP 40 g/kg group, samples with purple was PLP 20 g/kg group.

To comprehensively analyze the network of metabolic changes, we further conducted a PCA for the 10 metabolites included in the signaling network described in section 3.6: L(D)-arginine, 4-guanidinobutanoate, pantothenate, L-tryptophan, 2-phenylacetamide, 11-deoxycortisol, glycocholic acid, glycochenodeoxycholic acid, taurocholic acid, and protoporphyrin IX. As shown in **Figure 8A**, these metabolites were divided into three classes, indicated by the green bars. The control group was included in the first class. The second class contained all 80 g/kg PLP samples and one 40 g/kg PLP sample. Most of the 40 g/kg PLP samples, all 20 g/kg PLP samples and the ANIT group were included in the third class (**Figure 8A**). The heat map directly indicates changes in these samples. Specifically, samples in the control group were thoroughly divided from samples in the ANIT group, whereas the distance between the 80 g/kg PLP and ANIT groups was large. Moreover, 40 g/kg PLP was closer to the ANIT group than the 80 g/kg PLP group, and the 20 g/kg PLP was the closest to the ANIT group. This distribution indicated that the metabolites in the 80 g/kg PLP were more similar to those of the control group than those of the ANIT group. On the contrary, the metabolites of the 40 and 20 g/kg PLP groups were more similar to those of the ANIT group than those of the control group (**Figure 8B**).

## Discussion

### Therapeutic Efficacy and Possible Mechanism by Which PLP Affects Cholestasis shown in Previous Research

Cholestasis is the intrahepatic accumulation of potentially toxic bile acids that occurs in several liver diseases due to the obstruction or destruction of bile ducts (Serviddio et al., 2014). Because the outcomes of cholestasis are primary biliary cirrhosis and sclerosing cholangitis, this disease is invariably considered difficult to cure (Kim et al., 2000). At present, UDCA is recognized as a specific and potent treatment for cholestasis and is often combined with glucocorticoids (Buryova et al., 2013). However, the long-term administration of glucocorticoids may lead to several side effects. Additionally, UDCA is not consistently efficacious (Arenas et al., 2008). Thus, TCM is considered to provide a novel approach for treating cholestasis. Specifically, PLP is one of the most well known herbs in many Asian countries, such as China, Korea, and Japan. In the clinic, it consistently exhibits effects on liver disease, especially at high doses. In some cases, PLP has been used at doses of up to 200 g per day, which is ten times the conventional dosage, and continued to demonstrate positive and stable effects on cholestatic hepatitis without toxicity (Zhu and Wang, 2012). From a mechanistic perspective, we previously proved that paeoniflorin, the main active component of PLP, displayed its effect on cholestasis by reducing the production of ROS and NOX4 (Zhao et al., 2013). These findings partially reveal the mechanism of PLP because cholestasis progression is believed to not only relate to oxidative stress but also other important factors, such as the dysregulation of bile acid and inflammation. Currently, many studies have demonstrated that TCM for specific diseases regulates body function in various organs and tissues in a network manner (Li et al., 2007; Li and Zhang, 2013). Thus, metabolomics might help to identify the mechanism by which PLP affects cholestasis.

### Large Dose Application in Traditional Chinese Medicine

In this study, the maximum dose of PLP was 80 g/kg⋅days (as raw herb), which is high compared with the normal dose of this drug (15 g/kg⋅days). In fact, high doses of Chinese herbs have been widely applied since ancient times, and this approach remains interesting. For instance, the recorded doses of *Gypsum Fibrosum* applied in the *Yi Xue Zhong Zhong Can Xi Lu* (a medical book in Qing dynasty) reached up to 300 g per day for relieving fever. This dose is thirty times the currently used common dose (10 g per day; Peng, 2003). Furthermore, according to the *Li Ke Medical Records*, the dose of *Radix Aconiti Lateralis* Preparata used in Po-Ge-Jiu-Xin-Decoction was 500 g per day, which is ten times the regular dosage described in the Chinese Pharmacopeia. Several studies indicated that *Radix Aconiti Lateralis* Preparata at this dose exhibited remarkable therapeutic effects on severe shock and circulatory failure (Sun, 2007).

Our previous research revealed that high doses of PLP were not toxic (Wei, 2012). In this study, the weights of rats administered 80 g/kg PLP slightly decreased, but the serum indices and histological data suggested that the livers of rats in this group were more effectively protected. The effect of this high dose might be partially related to the chemical properties of paeoniflorin. Paeoniflorin protects tissues from cholestasis, liver fibrosis, acute hepatitis and non-alcoholic fatty liver disease (Zhao et al., 2013, 2015; Chen et al., 2015a; Zhang et al., 2015). However, the bioavailability of paeoniflorin is extremely low (Jiang et al., 2012). Therefore, the application of high doses may increase the level of paeoniflorin in the serum. In addition, we speculate that the action of high-dose PLP may mechanistically differ from that of low-dose PLP.

### Metabolites Characteristic of PLP Therapy in Cholestasis

Bile acids are recognized as crucial signaling molecules that regulate a variety of homeostatic mechanisms (Thomas et al., 2008). The TBAs in the blood are also reportedly commonly increased due to the impaired biliary excretion of bile acids during cholestasis (Chen et al., 2008). In this study, the levels of several bile acids, such as glycocholic acid, taurocholic acid and glycochenodeoxycholic acid, were significantly increased after ANIT treatment, which corroborates previous findings (Aoki et al., 2011). Notably, glycochenodeoxycholic acid and glycocholic acid are hydrophobic bile acids. Several studies have illustrated that the accumulation of several hydrophobic bile acids during cholestasis might result in hepatotoxicity via apoptosis and subsequent inflammation (Woolbright et al., 2015). Taurocholic acid is also one of the main bile acids for intrahepatic cholestasis and is believed to be involved in the pathogenesis of this disease by provoking a potent inflammatory response (Zhang et al., 2014; Woolbright et al., 2015). PLP (80 g/kg) markedly down-regulated the levels of these bile acids in the serum, which suggested that the observed prevention mechanism is closely related to bile acid synthesis and secretion.

In addition to bile acids, several representative amino acids, such as arginine and L-tryptophan, were also detected in this study. L-Arginine plays important metabolic roles in the formation of a number of important physiologic factors, including nitric oxide (NO), urea, creatine and even growth hormone release (Shao and Hathcock, 2008). A recent study also revealed that L-arginine protects rats from cholestasis via NO synthesis and attenuating oxidative stress (Ozsoy et al., 2011; Tain et al., 2013). L-Tryptophan is the precursor in serotonin synthesis. Serotonin is synthesized from L-tryptophan by the enzymes tryptophan hydroxylase and aromatic amino acid decarboxylase (AADC), and it regulates physiological functions in the hepatogastrointestinal tract (Lesurtel et al., 2008). Furthermore, Osawa et al. (2011) reported that L-tryptophan and its metabolite serotonin are involved in metabolic liver diseases, such as non-alcoholic fatty liver disease. Accordingly, we also observed a significant decrease in L-tryptophan in response to treatment with 80 g/kg PLP compared with ANIT rats.

### Possible Signaling Profile Associated with PLP Prevention in Cholestasis

In this study, bile acid secretion and amino acid metabolism were recognized as the main signaling network in cholestasis and PLP treatment (**Figure 5**). In cholestasis, impaired bile flow leads to the accumulation of bile acids in the liver, causing hepatocyte and biliary injury and inflammation (Li and Apte, 2015). The changes in several bile acids, such as glycocholic acid, taurocholic acid and glycochenodeoxycholic acid, constitute both the phenotype and cause of the progression of cholestasis. In our study, we also identified significant changes in these metabolites. In addition, several proteins and transporters in hepatocytes are specifically changed during cholestasis. Therefore, we measured the protein levels of BSEP, MRP2, and NTCP. NTCP is the intake pump that transports bile acid in the blood into hepatocytes. BSEP and MRP2 are the export pumps that transport bile acids from hepatocytes into the bile duct. Our data indicated that ANIT decreases the expression of BSEP, MRP2, and NTCP. It might be due to preventing the bile acid transporters in vesicles from being moved to the canalicular membrane. Moreover, the lower expression of transporters could further cause accumulation of bile acid. Treatment with PLP was able to significantly increase the lower expression of NTCP, BSEP, and MRP2 as well as bile acid such as glycocholic acid, taurocholic acid, and glycochenodeoxycholic acid. The result implicated that the therapeutic effect of PLP on cholestasis was probably associated with the bile acid secretion signaling pathway. Other signaling pathways, such as the generation of bilirubin and hormonogenesis, might also provide insight into cholestasis development and prevention. Notably, the increase in bilirubin is the most hallmark of cholestasis in both the clinic and laboratory research. We observed that the level of the upstream metabolite protoporphyrin IX was also increased in cholestasis. Interestingly, cholestasis might be relevant to the dysfunction of bilirubin metabolites, which is the sign of cholestasis. Changes in amino acids were also detected in our research. In several previous studies, amino acids were found to play important roles in liver injury and cholestasis. ROS, a key factor in hepatocyte apoptosis, was proven to be related to L-tryptophan overexpression (Nocito et al., 2007). Moreover, sulfur-containing amino acids, such as *S*-adenosylmethionine (SAM), are potential agents for cholestasis treatment. SAM positively regulates GSH, which is the most important antioxidant molecule in the liver (Jung, 2015). L-Arginine also efficiently treats cholestasis via NO synthesis and oxidative stress prevention (Ozsoy et al., 2011; Tain et al., 2013). Furthermore, amino acid metabolism is upstream of the KEGG pathway maps (01100, metabolic pathways), and several amino acids, such as L-arginine, L-tryptophan and 2-phenylacetamide, may ultimately regulate bile acid generation. Although this study did not conclusively identify the mechanism by which amino acids change and influence the downstream signaling pathway in cholestasis, it did initially depict that changes in amino acids are an important indicator of and reason for cholestasis progression.

## Conclusion

The serum biochemistry and histopathology data demonstrated that PLP exerts a significant anti-cholestasis effect. Specifically, a multivariate analysis detected differences in the metabolic profile in response to PLP treatment. Twelve metabolites, including glycocholic acid, taurocholic acid, glycochenodeoxycholic acid, L-tryptophan, protoporphyrin IX and pantothenate, were identified as markers of cholestasis and PLP treatment. The pathway and network analyses further indicated that ANIT-induced cholestasis and PLP treatment primarily affected bile acid secretion and amino acid metabolism, as indicated by significant changes in associated metabolites. Moreover, the significant changes in bile acid transporters expression also indicated that bile acid metabolism is involved in the therapeutic effect of PLP on cholestasis. PCA analysis confirmed the pharmacological changes induced by PLP at the metabolite level. According to the result above, the changes in metabolites and pathways, which are primarily related to bile acid secretion and likely related to amino acid metabolism, may partially clarify the therapeutic efficacy of PLP in treating cholestasis.

## Author Contributions

XM, Y-HC, and MN performed the experiments, analyzed the data, and wrote the manuscript. YZ, ZC, C-EZ, and J-YL collected and prepared samples. J-BW, L-FW, MG, S-ZW, and CC performed the analyses. LZ and M-QW amended the paper. Y-LZ and X-HX designed the study and amended the paper.

## Conflict of Interest Statement

The authors declare that the research was conducted in the absence of any commercial or financial relationships that could be construed as a potential conflict of interest.

## Footnotes

1 http://metlin.scripps.edu/

2 http://www.metaboanalyst.ca/

## ABBREVIATIONS

FcRnANIT: alpha-naphthylisothiocyanate
ALP: alkaline phosphatase
ALT: alanine aminotransferase
AST: aspartate aminotransferase
BSEP: bile salt export pump
DBIL: direct bilirubin
MRP2: multidrug resistance-associated protein 2
NTCP: Na^+^/taurocholate cotransporting polypeptide
PCA: principal component analysis
PLP: *Paeonia lactiflora* Pall.
PLS-DA: partial least-squares discriminant analysis
Q-TOF: quadruple-time-of-flight
TBA: total bile acid
TBIL: total bilirubin
TCM: traditional Chinese medicine
UDCA: ursodeoxycholic acid
UHPLC: ultra high performance liquid chromatography.
